# Aeromonas and Plesiomonas

**DOI:** 10.3390/microorganisms13030569

**Published:** 2025-03-03

**Authors:** Troy Skwor

**Affiliations:** School of Biomedical Sciences and Health Care Administration, University of Wisconsin-Milwaukee, Milwaukee, WI 53211, USA; skwor@uwm.edu

This Special Issue was designed to highlight some of the research presented at the 13th International Symposium on *Aeromonas* and *Plesiomonas* in Wroclaw, Poland. Both bacterial genera are ubiquitous in aquatic environments and are associated with diseases of cold- and warm-blooded animals, including humans. This co-existence in various ecosystems might explain the significant acquisition of horizontal genes acquired by *Pleisomonas shigelloides* from *Aeromonadaceae*, many of which became part of the core genome of *Pleisomonas* [1]. Furthermore, its ubiquitous presence in numerous ecosystems, rich diversity of mobile genetic elements [2], and capability of interspecies genetic exchange [3] has identified *Aeromonas* spp. as a global One Health indicator species to monitor antimicrobial resistance [4,5]. This Special Issue expanded upon our understanding of this genome plasticity and its role in disseminating antimicrobial resistance (AMR) within *Aeromonas,* as well as a unique approach combating multi-drug resistant (MDR) infections.

Human clinical disease manifestations associated with *Aeromonas* spp. are more commonly identified as gastroenteritis among children, especially living in heavily populated cities with poor water quality and sanitation [6], and wound infections. Among the latter infections, fatalities can arise when they progress to bacteremia and necrotizing fasciitis. In this Special Issue, Huang et al. provided valuable insights on the details of and treatments associated with 15 cases of *Aeromonas*-induced necrotizing fasciitis (NF). Of the 188 confirmed cases of necrotizing fasciitis within a coastal tertiary hospital, 12% were caused by *Aeromonas* spp. Ineffective empirical antimicrobial use of the commonly prescribed vancomycin and ceftriaxone was strongly associated with mortality in 75% of the *Aeromonas*-induced NF cases. The intrinsic resistance of *Aeromonas* to vancomycin, along with 18.8% of the isolates associated with NF cases exhibiting resistance to the third-generation cephalosporin ceftriaxone, warrants reconsideration of the drugs prescribed during empiric antimicrobial therapy.

Another article in this Special Issue took a different approach to combat bacterial infections. Guerra et al. repurposed fenofibrate, an FDA-approved drug for treating hypercholesterolemia and hyperlipidemia, as a potential therapeutic agent against bacterial infections. While fenofibrate had minimal effects on planktonic MDR *Aeromonas* growth, this drug appeared to stimulate antimicrobial properties in macrophages that corresponded to reduced bacterial burdens, although within a narrow concentration range. Considering the growing threat of AMR and the lack of new antibiotics, alternative therapeutic approaches must continue to be explored.

The remaining research articles in this Special Issue provide novel insights into the plasticity of the *Aeromonas* genome. Skwor et al. provided the first report of a clinical strain of *Aeromonas hydrophila* encoding *bla*_SFO-1_ within a MDR plasmid. This finding supplements previous reports of *bla*_SFO-1_ encoding *Aeromonas* strains within stray dogs in Afghanistan and wastewater isolates in China, stressing the One Health interconnectedness of antimicrobial resistance. The authors identified that 25% of the 328 kb plasmid displayed nearly 100% homology with *Enterobacterales* members, specifically *Klebsiella* spp. and *Enterobacter.* The remaining 75% of the plasmid was homologous with a clinical isolate of *A. caviae*, thus further highlighting the interspecies genomic exchange within this genus.

The plasticity and continual evolution of the *Aeromonas* genome was further described by Baltazar-Cruz et al. They identified an *Aeromonas* isolate from the stool of a patient with gastroenteritis. A multi-locus phylogenetic analysis of fifteen housekeeping genes failed to identify a species for *Aeromonas* sp. 3925. Average nucleotide identity with *A. media* and *A. rivipollensis* exhibited <95% similarity after whole genome sequencing, resulting in a new genomospecies, *paramedia.* Furthermore, this article was the first to identify a chromosomal class 4-like integron integrase in *Aeromonas.* An amino acid comparison exhibited a high degree of similarity to other γ-proteobacteria, including members of *Enterobacterales,* further demonstrating the common genetic exchange across species.

Beyond interspecies genetic exchange, Marcoux et al. identified a high frequency of recombination between two separate plasmids, pAsa5 and pAsa8, within an *Aeromonas salmonicida* subsp. *salmonicida* strain. pAsa5 was previously known to carry a type III secretion system (T3SS), which is essential for virulence in fish, surrounded by insertion sequences. The abundance of insertion sequences within this plasmid has resulted in multiple rearrangements within the plasmid with subsequent T3SS component loss [7]. Marcoux et al. expanded upon our understanding of insertion sequence recombination by conjugating a donor strain carrying pAsa8 with various antimicrobial resistance genes (ARGs) within a Tn*1721* transposon to a pAsa5 encoding recipient. The authors identified fusion of the two plasmids at a common 22-nucleotide sequence of a tyrosine recombinase site among multiple transconjugants.

Together, these research articles elaborate upon complexities associated with multi-drug resistance among *Aeromonas* spp., further stressing the need to address AMR from a One Health perspective. I wish to give a special thanks to my co-editor Dr. Marta Kaszowska, who was instrumental in organizing the conference associated with this Special Issue. Thanks also to the authors of these research articles for aiding in the continual understanding of these complex organisms. Lastly, I wish to thank the journal *Microorganisms* for the opportunity to focus a Special Issue on this topic.
